# Negative Symptoms of Schizophrenia: New Prospects of Cariprazine Treatment

**DOI:** 10.17650/2712-7672-2020-1-2-43-51

**Published:** 2020-12-04

**Authors:** Aleksandr M. Reznik, Aleksandr L. Arbuzov, Sergey P. Murin

**Affiliations:** Medical Institute of Continuing Education of «Moscow National University of Food Production»; Mental-health Clinic No. 1 named after N.A. Alexeev; Mental Health Clinic No. 5

**Keywords:** cariprazine, negative symptoms, schizophrenia, mental disorders, treatment, карипразин, негативные симптомы, шизофрения, психические расстройства, лечение

## Abstract

**Background.:**

Cariprazine is a new piperazine derivative atypical antipsychotic, like aripiprazole and brexpiprazole. It has been approved for treating schizophrenia in many countries and has recently been included on the List of Essential Medicines in Russia. Unlike most other atypical antipsychotics, it shows high *in vivo* occupancy of dopamine D2 and D3 receptors at clinically relevant doses. In animal models, cariprazine has demonstrated dopamine D3 receptor-dependent pro-cognitive and anti-anhedonic effects, suggesting its potential for treating negative symptoms. This review summarizes the efficacy of cariprazine in the treatment of negative symptoms of schizophrenia.

**Methods.:**

A literature search of databases covering international and Russian journals, for articles published between 1st January 2010 and 1st June 2020.

**Results.:**

Cariprazine demonstrated at least comparable efficacy in the treatment of schizophrenia symptoms to active comparators including risperidone, olanzapine or aripiprazole. The drug has a good safety profile. It appeared to be associated with a lower risk of metabolic syndromes and most extrapyramidal symptoms. The positive effect of cariprazine on the negative symptoms of schizophrenia may be associated with the elimination of secondary negative symptoms. However, of all the atypical antipsychotics to date, only cariprazine has a convincingly, methodologically robust proven advantage over risperidone in eliminating the predominant negative symptoms of schizophrenia. Yet only four studies have investigated the effect of cariprazine on the negative symptoms of schizophrenia. There is a lack of research into its direct impact on emotional-volitional disorders, anhedonia, cognitive symptoms and personality changes. However, there is evidence to suggest cariprazine is effective in treatment-resistant cases, but this requires further confirmation.

**Conclusion.:**

Cariprazine is an effective and well-tolerated agent for the treatment of schizophrenia and may be effective in cases where other antipsychotics have failed. Cariprazine has been shown to have a positive effect on negative symptoms. Further studies are needed to collect more data on long-term treatment of schizophrenia and especially negative symptoms.

Emotional-volitional disorders were first identified as manifestations of a primary mental defect and early dementia (dementia praecox) by Emil Kraepelin at the end of the 19th century [1]. Other seminal research of the time, from the highly influential neurologists Sir John Russell Reynolds (1861) [2] and John Hughlings Jackson (1884) [3], advanced the notion of positive and negative symptoms of neurological disease. Since then, over a hundred years of study have shown that negative symptoms of schizophrenia differ from other symptoms (psychotic, cognitive, affective), both in patients with early psychosis and in the chronic stages of the disease. These negative symptoms affect quality of life, recovery and general functioning [4,5]. In recent years, an international consensus has been reached on negative symptoms, proposing two large clusters: apathy / abulia, which includes direct abulia, anhedonia and decreased social activity, and decreased expressiveness, which includes poverty of speech and affective flattening [6-8]. The experiences of positive symptoms at different stages of schizophrenia are very diverse and have generated much debate regarding diagnostic signs, the definitions of the disease and its variations [6,9-12]. According to Smulevich et al. (2020), interactions between negative and positive symptoms act as 'transformers' that modify the characteristics of originally nosologically neutral disorders [13]. In addition, negative disorders create a significant burden of disease and persist during periods of remission when positive symptoms have been greatly reduced. Ultimately, negative symptoms are the greatest determinants of poor social integration and decreased functioning, which deeply affect all aspects of patients' daily lives. They can also delay entry into specialized care [11,14-20].

Negative symptoms rarely appear in isolation. Negative changes arising during the prodromal stage are usually transformed by constitutional chromosome anomalies or previously latent personality issues. This can result in different variants of personal distortion, the extreme forms of which are the syndromes of expansive or defensive schizoidia. During disease stabilization, negative symptoms combine with residual positive symptom complexes, creating psychopathological formations characteristic of schizophrenia and distinct from other mental disorders. Moreover, positive symptoms lose their intensity and other qualitative characteristics over time and, thereby, expand the negative component of the general psychopathological syndrome [13].

Negative symptoms of schizophrenia have an enduring nature with a broad range of consequences. They present with irregular severity, exacerbate other psychopathological symptoms, can affect all aspects of patient functioning throughout the course ofthe disease and have a particular low sensitivity to treatment. Negative symptoms therefore began to be considered as a distinct area for investigation with special pathophysiological and therapeutic consequences [6,12]. Early treatment can reduce disease progression and improve outcomes [20]. However, knowing when to begin treatment can be challenging, because the disease often develops gradually and the symptoms form various clinical combinations. They are often difficult to identify or distinguish from symptoms of other clinical conditions, especially the depressive and cognitive symptoms [21-23]. If negative symptoms appear much earlier than the presentation of psychosis, there is a high probability that the disease will take a continuous course with poor prognosis [24-28]. The predominance of negative symptoms without noticeable psychotic signs significantly complicates diagnosis, leading to an increased duration of untreated psychosis [29], closely associated with a worse functional outcome [30]. However, even with accurate diagnosis via modern psychometric instruments and timely initiation of treatment, there is not an equal chance of reducing the severity positive and negative symptoms, since the latter are much less sensitive to any modern therapy [6,31,32].

Both first- and second-generation antipsychotics demonstrate good efficacy in eliminating or ameliorating the positive symptoms of schizophrenia [33,34]. Direct or indirect anti-negative effects are seen by a decrease in the score on negative scale of the Positive and Negative Syndrome Scale (PANSS) [35]. There is also a significant increase in the frequency of the most severe disease outcomes in patients who have not received treatment for a long time [20]. However, for a long time, the lack of progress against florid symptoms of schizophrenia outweighed other effects of antipsychotics. There has been an urgent need to develop means of prevention and treatment of negative symptoms, restoring psychosocial, family and professional functioning, improving quality of life while simultaneously minimizing the side effects of psychopharmacotherapy [33,34].

The use of second-generation antipsychotics has led to a significant decrease in neurological side effects, enhancing the efficiency of correction of affective disorders, improving medication compliance and increasing patients' social integration. It was originally assumed that first-generation atypical antipsychotics would be effective in treating primary negative symptoms and second-generation antipsychotics have shown themselves to be more effective [34]. However, in recent years, it has increasingly been suggested that these drugs did not change path of development of the disease or general prognosis [36-38]. A meta-analysis comparing first- and second-generation antipsychotics found that only four second-generation drugs (amisulpride, clozapine, olanzapine and risperidone) were more effective than first-generation drugs in treating negative symptoms (effect sizes from -0.13 to -0.32). The other five second- generation antipsychotics (aripiprazole, quetiapine, sertindole, ziprasidone and zotepine) did not show any significant effects [39]. It has been questioned whether the efficacy in treating negative symptoms can be considered the main component of antipsychotic atypicality [38]. In a second meta-analysis, antipsychotics showed better efficacy in treating negative symptoms than placebos. However, the effect size for negative symptoms (-0.39) was smaller than for general symptoms (-0.51) or productive symptoms (-0.48) [39]. It should be noted that most studies included in these meta-analyses examined patients with predominantly positive symptoms, so some improvement may have been associated with changes in other areas, e.g., reduced acuity of psychosis, decreased depression or neurological complications of pharmacotherapy [38]. The treatment of negative symptoms remained the main unmet need [38].

To some extent, raised expectations of second- generation antipsychotics were associated with their more complex and multimodal neuroreceptor effects. In addition to the usual D2-blocking action of classical antipsychotics, second-generation antipsychotics include affinity for D3 receptors, selective effects on various dopamine pathways, antagonism of several serotonin receptors and blocking of serotonin reuptake [35]. Over the past 15 years, the D3 receptor has been a potential neurochemical target for the treatment of negative, cognitive and emotional symptoms associated with schizophrenia, due to its predominant expression in the mesolimbic pathway [40-45]. Moreover, D3 activity appeared to be associated with the regulation of dopamine synthesis and release [46-48]. Antagonism of the D3 receptor has been suggested to enhance dopaminergic and cholinergic neurotransmission in certain brain areas, such as the prefrontal area [49,50]. In addition, targeting the D3 receptor is driven by the need to develop a new drug that will not cause psychopathological, neurological and autonomic side effects characteristic of D2 antipsychotics.

Further search for molecules with affinity for the D3 receptor led to the synthesis of a new active and strong partial antagonist of D3 and D2 receptors and a partial 5-HT1A agonist [44,51]. A new drug with the chemical formula trans-N- (4- (2- (4- (2,3-dichlorophenyl) piperazin-1-yl) ethyl) cyclohexyl) -N ', N'-dimethylamide was registered with the international non-proprietary name 'cariprazine'. Chemically, it belongs to piperazine derivatives, similar in structure to aripiprazole and brexpiprazole [33]. Its brand names are Vraylar® (USA) and Reagila® (Europe) [52]. In contrast to other antipsychotics, cariprazine in clinically relevant doses demonstrates high in vivo occupancy of both D2 and D3 receptors [51,53]. In animal models, it demonstrated a D3-dependent positive effect on cognitive functions and an antidepressant-like effect on manifestations of anhedonia, which indicate its potential for treatment of negative symptoms [54,55]. Cariprazine and aripiprazole both act as partial agonists of D2 and D3 receptors, but cariprazine is much more selective with respect to D3 than to D2 [43]. It is inferior only to clozapine in terms of its affinity for D3 receptors. Various studies on cloned human receptors in vitro have shown that the affinity of cariprazine for D3 receptors is six to 10 times that for D2 receptors [43,56-58]. In a recent trial using positron emission tomography in healthy adults, cariprazine showed significant occupancy of the D3 dopamine receptor (N60%) even at a dose of 1 mg/day [53]. In patients with schizophrenia at a dose of 1.5 mg/day, the occupancy rate of D2 and D3 receptors exceeded 69-75% [59]. The fact that cariprazine is a weak partial agonist rather than a full antagonist of D2 and D3 receptors makes it possible to achieve a very high occupancy rate (approaching 90-100%) during treatment with therapeutic doses without th development of pronounced extrapyramidal symptoms. This is an advantage over use of full D2 antagonists, either classical or atypical antipsychotics, as they lead to the development of severe extrapyramidal symptoms and even absolute akinesia [50,60]. It is hoped that cariprazine will be effective in cases where other antipsychotics have only achieved 60-80% of the occupancy rate of D2 receptors, which is insufficient to obtain a therapeutic effect [50,60]. Moreover, long-term administration of cariprazine can increase the density of D3 receptors in a number of brain regions. In untreated schizophrenic patients, the expression of D3 receptors (formation on the cytoplasmic membrane) is usually initially reduced in contrast to the increased expression of D2 receptors. Therefore, upregulation of D3 receptors (regulation aimed at increasing receptor density) is likely to be a beneficial effect of cariprazine [61]. The indirect administration of cariprazine leads to upregulation and functional activity of NMDA receptors which is not observed in other non-clozapine antipsychotics [61]. NMDA receptors can be considered the most important therapeutic target in schizophrenia, since hypofunction indirectly causes disorders in the dopaminergic and serotonergic systems of the brain. Drugs affecting this, including cariprazine, are therefore highly sought [50].

As a partial agonist with receptor selectivity, cariprazine increases the activity of systems that are not stimulated enough by internal agonists and prevents excessive and harmful stimulation by an increased level of endogenous agonist in another system. It predominantly suppresses the excessive spontaneous activity of mesolimbic dopaminergic neurons, which determines its general and selective antipsychotic activity, and increases the activity of neurons in the mesocortical tract, which provides its possible anti-negative and pro-cognitive effects [58]. It also has a slight impact on the dopaminergic neurons of the nigrostriatal pathway and therefore has a low risk of provoking extrapyramidal symptoms, akathisia and tardive dyskinesia. It only weakly affects the receptors of the tuberoinfundibular pathway, which explains the low risk of hyperprolactinemia and sexual dysfunction [50,62].

In relation to serotonin receptors, Cariprazine is an agonist of 5-HT1A, a strong antagonist of 5-HT2B, a moderate antagonist of 5-HT2A and a weak antagonist of 5-HT2C [43,57,63]. These receptor properties increase the levels of dopamine and noradrenaline in the prefrontal cortex and the level of dopamine in the nigrostriatal and tuberoinfundibular pathways. This further reduces the probability of extrapyramidal symptoms, hyperprolactinemia, neuroleptic depressions, antipsychotic-induced negative disorders and cognitive deficiency [50,57].

Another advantage of cariprazine is the absence of antagonism for M3 cholinergic (muscarinic) receptors, as well as a low affinity for HI histamine and 5-HT2C serotonin receptors. This predetermines an almost complete absence of effects such as excessive sleepiness, increased appetite and weight gain, and a low risk for metabolic disorders and diabetes mellitus [50,57,64,65].

In summary, the predominant effect of cariprazine on D3 receptors in combination with partial agonism gives reason to expect a beneficial effect on the negative, cognitive and depressive symptoms of schizophrenia [33,42,43,55], a therapeutic effect in recalcitrant cases [33,60] and low risk of adverse effects [33].

Several randomized clinical trials have demonstrated its superiority over placebos in various doses (from 1.5 to 9 mg/day) on the PANSS and the Clinical Global Impressions Scale [57,62,66-68]. However, it is no more effective on acute psychotic symptoms than other antipsychotics, for example, risperidone [62].

In randomized clinical trials of cariprazine in long-term maintenance therapy (with recommended doses of 1.5 to 6 mg/day), it performed twice as well as a placebo for anti-relapse activity [65], safety and high tolerability [69,70]. It has also been shown to be superior to risperidone in addressing negative and affective symptoms and cognitive impairments, and positive effects on quality of life [71,72]. Good effects were also obtained via lower doses of cariprazine. However, pronounced differences in the effect on negative symptoms were only observed with long-term therapy, i.e., longer treatment than usual may be necessary to assess the effect on positive symptoms [71].

A meta-analysis of 21 randomized clinical trials found that of all non-clozapine antipsychotics, only cariprazine compared favourably with risperidone and was statistically superior in schizophrenic patients with a predominance of negative symptoms [38]. However, these findings were based on one large trial sponsored by the pharmaceutical company that produces cariprazine [73]. In relation to all other atypical antipsychotics, only an advantage over the classical antipsychotic (haloperidol) has been demonstrated. Pairwise comparisons of other second-generation non-clozapine antipsychotics have yielded conflicting results; sometimes the sample sizes were not large enough to draw firm conclusions [38].

Decrease in severity of negative symptoms over time via cariprazine is observed both in clinically stable patients with predominantly negative symptoms, and in patients with a worsening of the course of disease [73]. For example, at a dose of 4.5-6.0 mg once a day for six weeks was effective in treating moderate to severe negative symptoms without pronounced positive symptoms [73]. This trial included patients with predominantly negative symptoms for at least six months, scoring at least 15/24 on the negative symptom scale of the PANSS, or four or more on two of the three main negative disorders (flattening of affect, passive-apathetic social withdrawal, lack of spontaneity and fluidity of conversation). For both doses of cariprazine (3 and 4.5-6.0 mg, respectively) compared with placebos, the proportion of patients who met the response criteria for the PANSS factor score for negative symptoms (PANSS-FSNS) was significantly higher, including on the following items: flattening of affect (N1), emotional disengagement (N2), insufficient rapport (N3), passive-apathetic social withdrawal (N4), lack of spontaneity and fluidity of conversation (N6), motor retardation (G7) and active social avoidance (G16) [74]. In particular, cariprazine resulted in a significantly more pronounced decrease in the PANSS-FSNS score by the 26th week of treatment than in the risperidone group (-8.9 vs. -7.44; p = 0022; effect size -0.31).

In contrast to cariprazine, there were no statistically significant differences in PANSS-FSNS scores for risperidone and aripiprazole compared with placebos [38]. It was shown indirectly that improvement in patients with a predominance of negative symptoms did not depend on the improvement of other (positive, depressive, extrapyramidal) symptoms [73]. This latter finding is very important, since the improvement of negative symptoms is often secondary to effects in other psychopathological domains, which makes it difficult to identify the specific effects of treatment on primary negative symptoms.

Finally, there is interest in the efficacy of cariprazine in relieving symptoms and restoring social functioning in patients who received no treatment for many years and had both persistent positive and severe negative manifestations of schizophrenia [38]. Cariprazine is much less likely than aripiprazole to cause exacerbation of productive psychopathological symptoms (delirium, hallucinations), agitation, anxiety or insomnia at the beginning of therapy, because it has lower internal agonistic activity on the D2 receptor [33].

Due to rather high neuroreceptor selectivity compared with aripiprazole and brexpiprazole, cariprazine has lower potential for causing metabolic side effects and has improved tolerability and safety. In particular, it is less likely to cause weight gain, hyperlipidaemia, hypertriglyceridemia, hypercholesterolemia or hyperglycaemia and less likely to lead to the development of type two diabetes or metabolic syndrome. Levels of metabolic side effects are the same as those from a placebo [57,65,68]. The low frequency of metabolic side effects and the probability of sedation means that it compares favourably with other second-generation antipsychotics. Among extrapyramidal symptoms arising from cariprazine, akathisia is most often noted, which is explained by the mechanism of effect of the drug [33]. However, the increased probability is only small compared with placebos, and less than in aripiprazole. Cariprazine-induced akathisia is usually not severe [65].

In conclusion, cariprazine has the potential to be good addition to existing treatments for schizophrenia and other primary psychotic disorders. It has proven remitative and anti-relapse antipsychotic activity, can eliminate prevailing negative symptoms, shows efficacy independently from other antipsychotics and has a good safety profile. However, its effects on certain domains of negative disorders (abulia, anhedonia, decreased social activity) remain insufficiently studied. Further research is needed to examine the effects of a new antipsychotic on complex psychopathological syndromes, including positive, negative, cognitive and affective symptoms at different stages of schizophrenia.

## Authors contribution

Alexandr M. Reznik: planning, searching, retrieval and selection of data, text preparation. Alexandr L. Arbuzov: searching and retrieval of data, text preparation. Sergej P. Murin: searching and retrieval of data. Alexej V. Pavlichenko: methodology, editing.

## Funding

The review was carried out without additional funding.

## Conflict of interest

The authors declare no conflicts of interest.
